# Acute Viral Pericarditis Complicated by Cardiac Tamponade as a Result of COVID-19

**DOI:** 10.7759/cureus.36695

**Published:** 2023-03-26

**Authors:** Inderpal Singh, Jordan Swisher, Harish Gidda, Bola Nashed, David Rodriguez

**Affiliations:** 1 Department of Internal Medicine, Ascension St. John Hospital, Detroit, USA; 2 Department of Cardiology, Ascension St. John Hospital, Detroit, USA

**Keywords:** sars-cov-2, cardiac arrest, cardiogenic shock, covid-19, pericardial effusion. cardiac tamponade

## Abstract

Severe acute respiratory syndrome coronavirus 2 (SARS-Cov-2) and coronavirus disease 2019 (COVID-19) predominantly cause respiratory symptoms but cardiovascular complications from COVID-19 have been documented in the literature. Acute pericarditis has been known to be caused by COVID-19 but severe cardiac complications, such as cardiac tamponade, have rarely been reported. Early diagnosis and treatment with pericardiocentesis are imperative, as this can improve patient outcomes.

A 56-year-old female presented with chest pain and recurrent episodes of presyncope. The patient tested positive for SARS-Cov-2 through a polymerase chain reaction (PCR) test. The patient was hypotensive on arrival and the initial workup with electrocardiogram was significant for sinus tachycardia with low voltage QRS complexes in the precordial and limb leads. A transthoracic echocardiogram was also done and showed a large circumferential pericardial effusion with chamber collapse of the right atrium and right ventricle during diastole indicative of tamponade physiology. The patient's clinical course was complicated by pulseless electrical activity cardiac arrest during which a pericardiocentesis was done. One hundred (100) mL of serous pericardial fluid was drained and a return of spontaneous circulation was obtained after roughly 10 minutes of cardiopulmonary resuscitation. Further infectious and noninfectious workups, including malignant and rheumatologic etiologies for acute pericarditis, were negative. The patient was subsequently treated with high-dose non-steroidal anti-inflammatory drugs (NSAIDs) and colchicine for viral pericarditis. The patient's clinical course improved, and the patient was subsequently discharged after a prolonged hospital course to a subacute rehabilitation facility to undergo physical therapy.

## Introduction

Severe acute respiratory syndrome coronavirus 2 (SARS-Cov-2) and coronavirus disease (COVID-19) predominantly cause respiratory disorders and illnesses, including low or high-grade fever, cough, fatigue, dyspnea, and acute respiratory distress syndrome [1]. Although relatively rare, direct and indirect cardiovascular complications from COVID-19 have been documented [2]. Acute pericarditis is a disease that can be commonly caused by viral infections, including COVID-19. Acute pericarditis is a known complication of COVID-19 infection, but acute pericarditis complicated by cardiac tamponade is a relatively rare clinical entity [3]. Here, we present a case of acute viral pericarditis complicated by large pericardial effusion and tamponade as a result of COVID-19.

## Case presentation

Informed verbal consent was obtained from the patient. A 56-year-old female with a past medical history of hypertension and generalized anxiety disorder presented with a four-day history of chest pain, along with dizziness and recurrent episodes of presyncope. The patient had been reporting poor oral intake for roughly one week prior to symptom onset. On arrival, initial vital signs were significant for sinus tachycardia, with a rate of 106 beats/minute, and hypotension, with a systolic blood pressure of 86 mmHg. On physical exam, the patient was alert, awake, and answering questions appropriately without conversational dyspnea. Dry mucous membranes were noted when the oral mucosa was examined. Upon auscultation of the chest, distal heart sounds were noted with no murmurs, rubs, or gallops. Lung sounds were heard in all lung fields on auscultation without evidence of rhonchi, wheezing, or rales. Pitting peripheral edema was noted in the lower extremities bilaterally up to the level of the knees. The initial laboratory workup is outlined in Table 1.

**Table 1:** Initial laboratory workup on admission COVID-19 - coronavirus disease 2019, PCR - polymerase chain reaction

Test	Results	Reference Range
White blood cell count	11.22	4.00-11.00 x 10^3^/uL
Hemoglobin	16.3	12.0-16.0 gm/dL
Creatinine	1.44	0.5-1.1 mg/dL
Blood Urea Nitrogen	37	6-23 mg/dL
Troponin-T	0.07	<0.05 ng/mL
Lactic Acid	4.1	0.5-2.0 mmol/L
COVID-19 PCR	Positive	Not Detected

**Figure 1:**
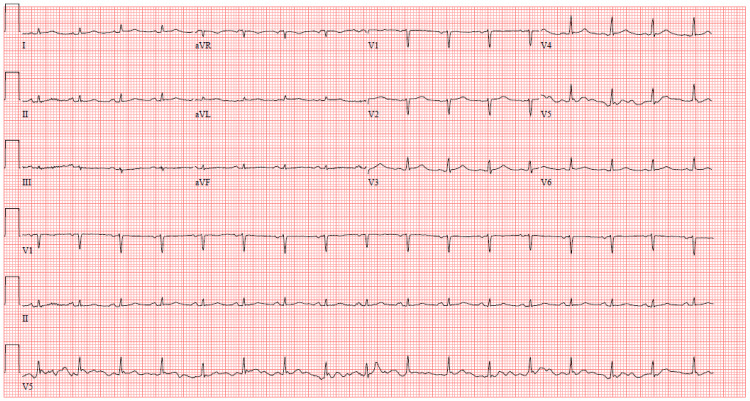
A 12-lead electrocardiogram showing sinus tachycardia with low voltage QRS complexes in both precordial and limb leads with electrical alternans

**Figure 2:**
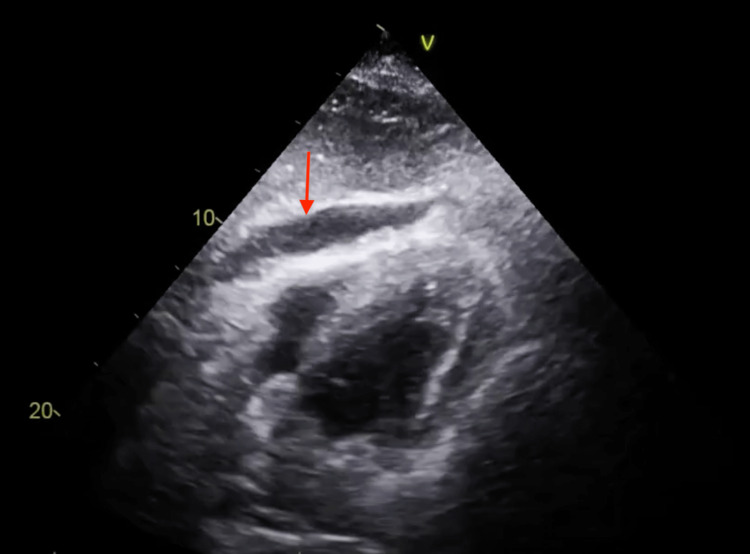
Echocardiogram subcostal view showing large pericardial effusion (arrow) with evidence of tamponade physiology

**Figure 3:**
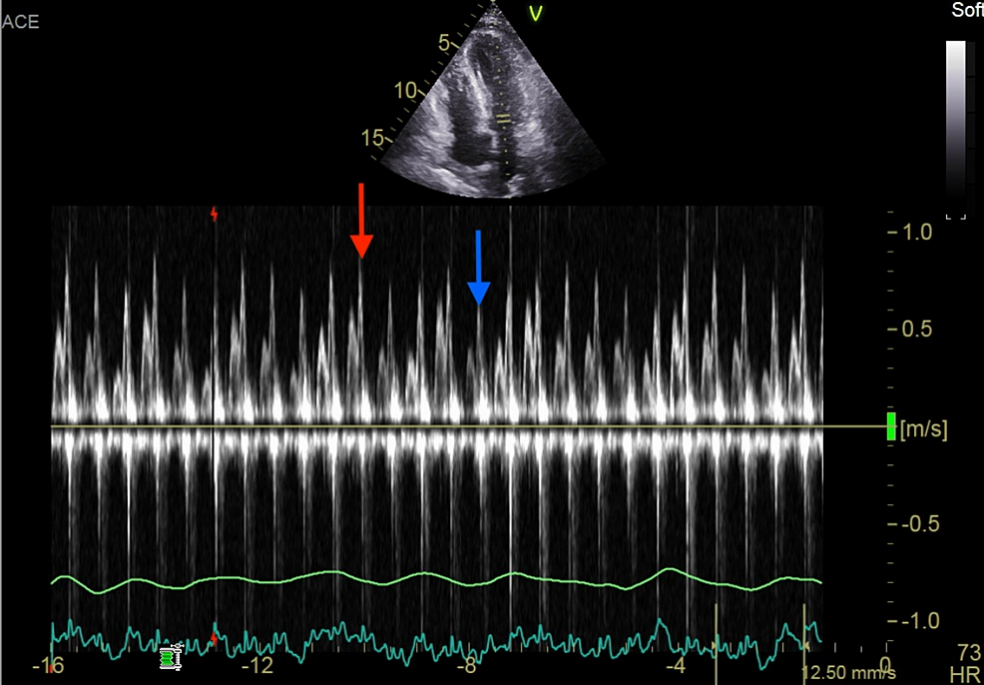
Echocardiogram Doppler view showing a variation of transvalvular flow velocity across the mitral valve The peak flow velocity during expiration is 0.9 m/s (red arrow) and 0.63 m/s during inspiration (blue arrow), which is a 30% decrease, a finding characteristic in cardiac tamponade.

Of note, the patient stated she was not vaccinated for COVID-19. The patient was fluid-resuscitated in the emergency department. Further workup, including electrocardiogram, was significant for sinus tachycardia with low voltage QRS complexes in both precordial and limb leads (Figure 1). A transthoracic echocardiogram was also done and was significant for a large circumferential pericardial effusion with coagulum predominantly surrounding the right ventricle, left ventricular apex, and distal left ventricular wall (Figure 2). Chamber collapse during diastole of the right atrium and right ventricle was also seen, indicative of tamponade physiology. Doppler transvalvular flow velocities across the mitral valve during respiration were also measured, and this showed a greater than 25% decrease in mitral flow velocity during inspiration, a finding that is seen in cardiac tamponade (Figure 3). An elevated pulmonary artery systolic pressure of 38 mmHg, increased inferior vena cava diameter of 2.6 cm, and elevated central venous pressure of 20 mmHg were also noted on the echocardiogram. The patient was admitted to the medical intensive care unit where the clinical status of the patient rapidly deteriorated whereby the patient required intubation and vasopressor support. The patient’s clinical course was complicated by pulseless electrical activity cardiac arrest during which a pericardiocentesis was performed and return of spontaneous circulation was obtained after roughly 10 minutes of cardiopulmonary resuscitation. One hundred (100) mL of serous pericardial fluid was drained during the procedure.

The patient’s course was further complicated by decreasing urine output and rising creatinine due to hemodynamic acute tubular necrosis. The patient was initiated on continuous renal replacement therapy at that time. Further workup to elucidate the etiology of pericardial effusion, as outlined in Table 2, including a respiratory viral panel for influenza virus, adenovirus, parainfluenza virus, and rhinovirus, among others was negative. Pericardial fluid analysis was done and showed a pericardial fluid to serum protein ratio of 1.08, along with an elevated pericardial fluid LDH of 259 units/L, which indicates an exudative effusion. Subsequent bacterial and fungal cultures of the pericardial fluid were negative for any growth. Cytopathology of pericardial fluid was negative for any evidence of malignancy. The rheumatologic workup was negative as well. Reverse-transcriptase polymerase chain reaction (RT-PCR) testing for SARS-CoV-2 in the pericardial fluid was also negative.

**Table 2:** Further infectious and rheumatologic workup to determine etiology for pericardial effusion HIV - Human immunodeficiency virus, Ag - Antigen, Ab - Antibody, PR3 - Proteinase 3, ANCA - Antineutrophil cytoplasmic antibodies, MPO - Myeloperoxidase, SSA - Sjögren’s syndrome-related antigen, SSB - Sjögren’s syndrome type B

Test	Results	Reference Range
HIV-1, HIV-2 Ag and Ab	Nonreactive	Nonreactive
Antinuclear Ab	Negative	Negative
PR3-ANCA	Not detected	Not detected
MPO-ANCA	Not detected	Not detected
Anti-Smith Ab	<0.2	0.0-0.9 AI
Ribonucleoprotein Ab	<0.2	0.0-0.9 AI
SSA Ab	<0.2	0.0-0.9 AI
SSB Ab	<0.2	0.0-0.9 AI
Smooth muscle Ab	Not detected	Not detected
Hepatitis A, B core, and C total Ab	Negative	Negative

Repeat echocardiography was done post pericardiocentesis with pericardial drain placement showing a remaining trivial pericardial effusion. The patient’s hemodynamics improved over the clinical course and vasopressor support was weaned off and the patient was extubated. The patient was also weaned off of renal replacement therapy over the course of the hospitalization, and the patient's kidney function improved back to baseline. The patient remained stable and was transferred out of the intensive care unit onto the general medical floor. The patient underwent physical and occupational therapy evaluation, which deemed the patient suitable for further rehabilitation at an outside facility. The patient was subsequently treated with high-dose non-steroidal anti-inflammatory drugs (NSAIDs) along with colchicine for acute viral pericarditis upon discharge. The patient was subsequently discharged to a subacute rehabilitation facility.

## Discussion

Acute pericarditis is a condition that involves inflammation of the pericardial sac [4]. The etiologies of acute pericarditis may be infectious due to either viral or bacterial causes, or noninfectious, where causes include inflammatory diseases, malignancy, or post-cardiac injury [5]. Viral causes are responsible for a vast majority of cases of acute pericarditis, with SARS-Cov-2 being a known cause in the literature [4]. Despite this, acute pericarditis with complications of severe cardiac disease, such as tamponade due to SARS-CoV-2, has rarely been noted in the literature [3].

The pathogenesis of COVID-19-induced acute pericarditis is unknown. Many mechanisms have been proposed, but it is thought that the mechanism of action of COVID-19-associated pericardial effusion and tamponade is through a systemic inflammatory response caused by acute viral illness [6]. In patients infected with SARS-CoV-2, an increase in serum pro-inflammatory cytokines was noted in the literature. These pro-inflammatory cytokines, including interleukin (IL) 1 beta, IL6, IL12, interferon-gamma (IFNγ), tumor necrosis factor-alpha (TNFα), and monocyte chemoattractant protein-1 among many others, potentially lead to activated T-helper-1 (Th1) cell responses [7-8]. This inflammatory response may subsequently lead to myocardial and perimyocardial inflammation leading to capillary leakage, causing the development of pericardial effusion. This is supported in the literature, as serum pro-inflammatory cytokine levels were seen to be elevated, more so in patients admitted to the intensive care unit (ICU), and evidence of significant inflammatory cell infiltration has been reported in the alveoli in patients with acute respiratory distress syndrome due to COVID-19 [9-10].

A second proposed mechanism of myocardial injury includes direct viral cytotoxicity caused by viral replication. This mechanism has been questioned in the literature as pathological findings have shown that no viral genomic fragments or intranuclear or cytoplasmic inclusions have been identified in the myocardium in patients with COVID-19 [6-9]. This is seen in the case presented here as a result of reverse transcription polymerase chain reaction (RT-PCR) testing of pericardial fluid post pericardiocentesis for SARS-Cov-2 was negative, implying no direct invasion or damage of the myocardium or pericardium is cause for this clinical picture. Despite no clear evidence of COVID-19 as the cause of acute pericarditis and cardiac tamponade in the patient presented in this case, it is suspected as such due to the negative infectious and non-infectious workup that was done, along with the patient’s positive result for SARS-Cov-2 polymerase chain reaction testing.

No current guidelines exist for the treatment of acute pericarditis with pericardial effusion specifically due to COVID-19. Due to the cytokine storm associated with severe COVID-19, specifically in patients admitted to the ICU, the use of corticosteroids has been shown to be of benefit, specifically in patients with inflammatory-induced lung injury [11]. In cases of cardiac tamponade, urgent pericardiocentesis is required to relieve the tamponade physiology and prevent hemodynamic compromise. In the case presented here, the patient's clinical status rapidly deteriorated and pulseless electrical activity cardiac arrest occurred, necessitating urgent pericardiocentesis. Once a return of spontaneous circulation was achieved and our patient was resuscitated, initial treatment with systemic corticosteroids was given. Once the patient improved and was transferred to the general medical floor, treatment with NSAIDs and colchicine was given until symptom resolution.

## Conclusions

COVID-19 is a viral illness that causes predominantly pulmonary manifestations. Although relatively rare, cardiovascular complications, such as myocarditis, acute coronary syndrome, or sudden cardiac arrest, may arise from this viral infection. Pericarditis associated with large pericardial effusion and tamponade is a severe potential complication from this condition and should be considered in patients with COVID-19 as early diagnosis and treatment with pericardiocentesis are critical to improve patient outcomes.

## References

[1] Minh LH, Abozaid AA, Ha NX (2021). Clinical and laboratory factors associated with coronavirus disease 2019 (Covid-19): a systematic review and meta-analysis. Rev Med Virol.

[2] Asif T, Kassab K, Iskander F, Alyousef T (2020). Acute pericarditis and cardiac tamponade in a patient with COVID-19: a therapeutic challenge. Eur J Case Rep Intern Med.

[3] Dweck MR, Bularga A, Hahn RT (2020). Global evaluation of echocardiography in patients with COVID-19. Eur Heart J Cardiovasc Imaging.

[4] Tonini M, Melo DT, Fernandes F (2015). Acute pericarditis. Rev Assoc Med Bras (1992).

[5] Imazio M, Gaita F, LeWinter M (2015). Evaluation and treatment of pericarditis: a systematic review. JAMA.

[6] Inciardi RM, Lupi L, Zaccone G (2020). Cardiac involvement in a patient with coronavirus disease 2019 (COVID-19). JAMA Cardiol.

[7] Huang C, Wang Y, Li X (2020). Clinical features of patients infected with 2019 novel coronavirus in Wuhan, China. Lancet.

[8] Zeng JH, Wu WB, Qu JX (2020). Cardiac manifestations of COVID-19 in Shenzhen, China. Infection.

[9] Xu Z, Shi L, Wang Y (2020). Pathological findings of COVID-19 associated with acute respiratory distress syndrome. Lancet Respir Med.

[10] Li L, Li L, Xiao L, Shangguan J (2018). Progranulin ameliorates coxsackievirus-B3-induced viral myocarditis by downregulating Th1 and Th17 cells. Exp Cell Res.

[11] Stockman LJ, Bellamy R, Garner P (2006). SARS: systematic review of treatment effects. PLoS Med.

